# Temporal changes in intensity and volume of external training loads during a 1 × 1 short-bout, small-sided games in elite youth soccer players

**DOI:** 10.1038/s41598-023-45277-y

**Published:** 2023-10-19

**Authors:** Jakub Kryściak, Maciej Tomczak, Tomasz Podgórski, Paweł Chmura, Marek Konefał, Jan Chmura, Tomas Maly, Toni Modric, Marcin Andrzejewski

**Affiliations:** 1 Department of Physiology and Biochemistry, Poznań University of Physical Education, 61-871 Poznan, Poland; 2 Department of Psychology, Poznań University of Physical Education, 61-871 Poznan, Poland; 3 Department of Team Games, Wroclaw University of Health and Sport Sciences, 51-612 Wrocław, Poland; 4 Department of Biological and Motor Sport Bases, Wroclaw University of Health and Sport Sciences, 51-612 Wrocław, Poland; 5 Faculty of Physical Education and Sport, Charles University, Prague, Czech Republic; 6 Faculty of Kinesiology, University of Split, 21000 Split, Croatia; 7 Department of Methodology of Recreation, Poznań University of Physical Education, 61-871 Poznan, Poland

**Keywords:** Biochemistry, Physiology

## Abstract

This study compared external training load (ETL) and its temporal changes across repetitions during a speed endurance production (SEP) training comprised of 1 × 1 short-bout, small-sided games (SSGs) in elite youth soccer players. Twenty U18 players were divided into two groups (SEP1 and SEP2) performing six 30 s and 45 s bouts of SSG (work-to-rest ratio 1:4) on a 10 by 15 m field. ETL was characterized by the total distance covered, Player Load, the total number of accelerations/decelerations, and their relative values (per minute). Significant overall decreases in the ETL parameter values across six repetitions in both SSG groups were observed relative to the measurement in the first set beginning in 3rd (SEP1) or 4th (SEP2) repetitions. Significant greater decreases in Player Load (F(5.90) = 2.99, p < 0.05, η P2 = 0.14), Player Load per minute (F(5,90) = 11.32, p < 0.001, η P2 = 0.39), total distance per minute (F(3.43,61.73) = 7.72, p < 0.001, η P2 = 0.30) and accelerations per minute (F(5,90) = 2.59, p < 0.05, η P2 = 0.13) were observed in the 30-s games than in the 45-s games. In conclusion, the use of SSGs in SEP training is associated with a decrease in the effectiveness of physical work performed across repetitions. In practice, due to the decrease in the measured ETL indicators already in the 3rd or 4th repetition (especially in the SEP1 group), the work-to-rest ratio could be increased from the applied 1:4 to 1:6.

## Introduction

Youth soccer is characterized by a wide spectrum of activities such as walking, low- and high-speed running, sprinting, jumping, kicking, accelerations and decelerations, rapid turns, ball control, dribbling, and tackling^1–6^. Therefore, match effort in soccer is characterized by both aerobic and anaerobic energy turnover and becomes increasingly physically demanding over time^7,8^. In recent years, no changes in the total distance covered by players during matches have been noted, but a significant increase in the number of intensive actions such as high-speed runs and sprints, as well as average distances covered during these activities, has been observed^9–11^.

Within a match youth soccer players (up to 18 years of age), cover total distance ranging from 6 to 11.5 km^1–3^. Depending on the age groups and speed thresholds used, youth soccer players cover 200 to 750 m of high-speed running, and 50 to 650 m of sprinting and also perform from 80 to even 155 accelerations and the same number of decelerations^1,3–5,12^. Sprint efforts do not occur singly, they are arranged in repeated-sprint sequences, usually consisting of two to four sprints lasting from 1 to 3 s, separated by no more than 60 s of recovery^13^. Therefore, to meet the match requirements, youth soccer players must have a high level of speed endurance capacity, which will ensure a high repeated-sprint ability, a quick recovery after each high-intensity effort, and maintain their performance throughout the match^14–17^.

Speed endurance training is defined as interval training of an intensity close to or above that eliciting maximum oxygen uptake (VO_2_max) and is divided into two subcategories: speed endurance maintenance (SEM) and speed endurance production (SEP) training^15,18–20^.

SEP training is used to improve players' ability to perform activities with maximal intensity for a relatively short period by positively affecting repeated-sprint ability and running economy during submaximal running^20–23^. SEP training is usually organized as interval runs (3 to 12 repetitions with a work-to-rest ratio between 1:4 and 1:6) lasting 10 s to 40 s with the inclusion or exclusion of relevant technical challenges performed at 70–100% of the maximum speed^19–21,24^.

The available literature is scarce concerning the use of small-sided games (SSGs) in SEP training^19,25,26^. In practice, it seems that SEP training can be effectively implemented in the SSG 1 × 1 format. As demonstrated by Kryściak et al.^26^ and Ade et al.^19^, short-bout 1 × 1 SSGs are characterized by very high physical (external training loads [ETL], including GPS metrics) and physiological (internal training loads [ITL], including heart rate, blood lactate concentration, and rate of perceived exertion) demands that meet the requirements of SEP training. In addition, 1 × 1 SSGs not only improve the physical performance of players but also affect technical skills, including the ability to dribble and one-on-one duels, which play a crucial role in the final score of a soccer match^27^. Due to the extremely high involvement of anaerobic glycolysis during short-bout 1 × 1 SSGs training^19,26^, the organism's homeostasis is disturbed, which may affect the decrease in the effectiveness of the work performed^14,15,17^. Important correlates of the effectiveness of work performed during soccer training are ETL parameters, such as Player Load (PL) and total distance covered (TD) (both expressed in relative units per minute) as well as acceleration (ACC) and deceleration (DEC) numbers^28^.

Due to the high application importance, the listed ETL indicators are tested often. However, as we showed above, the number of studies on the simultaneous, comprehensive analysis of the volume and intensity of loads in youth soccer players is limited^28^. In addition, these analyses do not apply to short-bout, 1 × 1 SSGs that can be used in SEP training.

Therefore, our study investigated how six successive 1 × 1 SSGs with different bout durations (30 s and 45 s) with the same work-to-rest ratio (1:4) affect work performed (measured by parameters characterizing the volume and intensity of ETLs) by youth soccer players. Secondly, we determined possible differences in the course of ETL changes resulting from SEP training in the form of 1 × 1 SSGs using bouts of different time durations in the group of U18 soccer players. In light of previous research, we assumed that SSGs with shorter bout durations are characterized by greater exercise intensity, which may result in a greater decrease in the efficiency of the training work performed by the youth soccer players.

## Methods

### Study design

**Figure 1:**
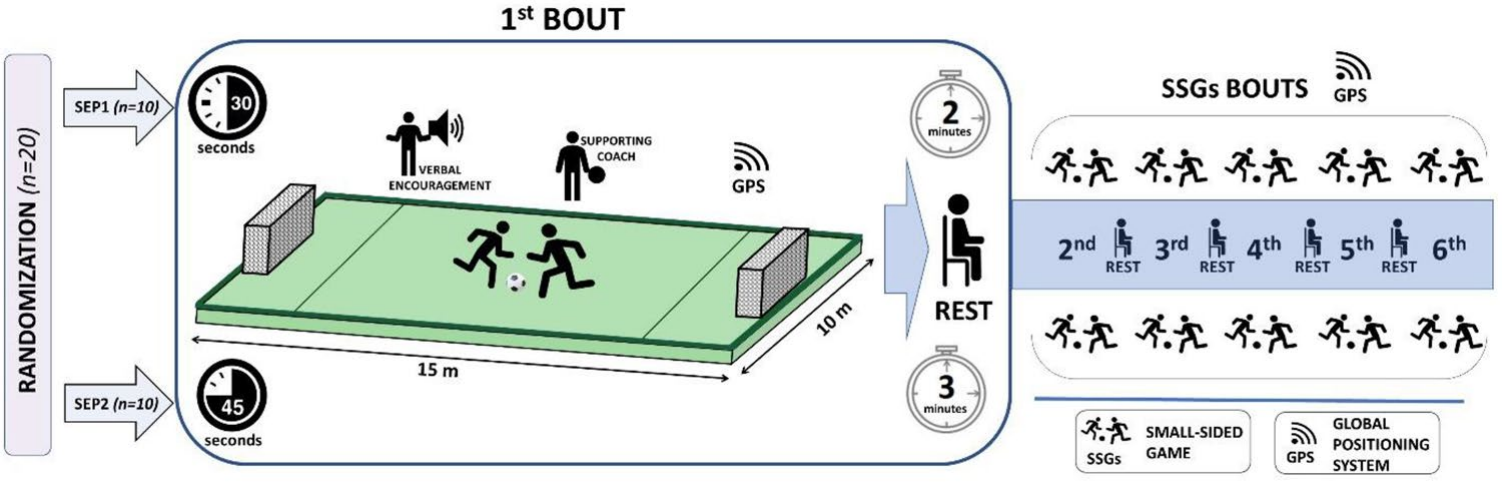
Study design. SSGs: small–sided games; SEP1: 6 × 30 s 1 × 1 SSGs; SEP2: 6 × 45 s 1 × 1 SSGs; GPS: Global Positioning System.

This research is a continuation of analyses on the physical and physiological characteristics of 1 × 1 short bout small-sided games initiated in the manuscript of Kryściak et al.^28^. The previous article deals with the analysis of internal training loads, while the current paper considers the effect of applied training on the parameters of external training loads. Therefore the primary aim of the study was to determine how SEP training conducted in the form of successive six 1 × 1 SSGs (Table 1) with different bout durations (30 s and 45 s) with the same work-to-rest ratio (1:4) affects work performed (ETL measured based on GPS metrics) by youth soccer players (Fig. 1). For this reason, twenty U18 players were randomly divided into two groups of 10, which performed six 1 × 1 SSGs with bouts lasting 30 s for the SEP1 group and 45 s for the SEP2 group. The athletes’ activity profiles (work performed) during the SSGs were measured using a portable GPS device (Catapult S5 Melbourne, Innovations, Australia).

**Table 1:** Characteristics of small-sided games used during the study.

1 × 1 SSG group	Bout duration [s]	Number of bouts	Passive recovery [s]	Work-to-rest ratio	Pitch dimensions WxL [m]
SEP1	30	6	120	1:4	10 × 15
SEP2	45	6	180	1:4	10 × 15

SSG: small-sided game; WxL: width and length; SEP1: 6 × 30 s 1 × 1 SSG; SEP2: 6 × 45 s 1 × 1 SSG.

### Participants

The study involved 20 players (age: 17.2 ± 0.83 years; body height: 178.2 ± 5.01 cm; body mass: 70.9 ± 5.43 kg; training experience 8.6 ± 3.25 years) of the U18 team, training at the academy of the soccer team from the highest league in Poland.

The research was carried out in a competitive period. In a standard microcycle during this training period, the players trained five times and played one official match at the highest level in Poland. The criterion for excluding an athlete from the study was a failure to complete the entire training protocol or incorrect recording of GPS data. Complete GPS data was obtained from all recruited participants; therefore, the results from all 20 youth soccer players were subjected to statistical analysis.

The participants, their parents or legal tutors, and coaches were fully informed about the study design and its requirements as well as the potential benefits and risks. Written, informed consent from all the research participants or legal tutors was obtained. The study was approved by the Research Ethics Committee (no. 20/2017) of the University School of Physical Education in Wroclaw and followed the institutional ethical requirements for human experimentation under the Declaration of Helsinki.

### Procedures

At the beginning of the study, the players were randomly divided into two groups (SEP1, n = 10; SEP2, n = 10). All players were familiarized with the experimental procedures and drills before data collection. The main part of the training, in which all players performed six bouts of 1 × 1 SSGs with passive recovery breaks, was preceded by a 20-min standardized soccer warm-up based on running at low intensities and dynamic stretching exercises followed by ball-specific stretching and a final 5-min ball possession activity to prepare for the study-specific task.

To meet the requirements of SEP training^21–23,26^, the duration of each repetition in the case of SEP1 group players was 30 s and in the SEP2 group was 45 s. The same work-to-rest ratio of 1:4 was used in the both tested group.

During the study, the players competed in unchanged pairs. To ensure the players maintained maximum intensity during the SSGs and remained committed to the training, coaches conducting the training provided regular verbal encouragement. The SSGs were conducted on a pitch with a synthetic surface with a dimension of 10 × 15 m surrounded by sideboards. The games were played with small goals but without goalkeepers. To ensure the quick pace of the games, the supporting coach passed the balls to the players as soon as a goal was scored or the ball went beyond the sideboards surrounding the pitch. The research protocol was conducted on the same day in the morning (10:00 to 14:00) in stable weather conditions (sunny, windless weather, and an ambient temperature of 10–12°C). The pitch surface was dry during all training sessions.

### ETL measurement

The variables characterizing the external work performed by players during the SSGs training were recorded using the Catapult S5 portable GPS with a sampling frequency of 10 Hz and using a 100 Hz triaxial accelerometer. According to the manufacturer's instructions, the device was activated 15 min before the start of the research protocol. To ensure the device worked properly and the players were comfortable wearing it, the GPS transmitters were fitted to the upper back of each player using a special harness. The reliability and validity of the Catapult S5 system have been previously confirmed^29^.

For the purposes of this study, four GPS variables characterizing the volume of ETL were recorded: total distance covered (TD, m), Player Load (PL, a.u.) and the total number of accelerations/decelerations (ACC/DEC, above 2 m·s^−2^, n).

PL is a measure from triaxial accelerometers in the GPS, calculated automatically using an established algorithm. It represents the sum of accelerations recorded in the anteroposterior, mediolateral, and vertical planes of movement. Previous research has shown that PL is a valid and reliable measure and can provide important insights into player physical activity^30,31^.

The number of ACCs was measured based on the changes in GPS speed and was defined as a change in speed for a minimum period of 0.5 s with an acceleration of at least 2 m·s^-2^. The same approach was used to determine the number of DECs.

To obtain the intensity characteristics (external intensity measures) of the SSGs used in the SEP training, all variables recorded were also reported in relative terms (per minute). The maximum velocity (MV, km∙h^-1^) achieved by the players in each game was also measured.

### Statistical analysis

To compare the specified groups in terms of changes in the studied parameters over time, we used a two-factor ANOVA with one between-group factor (two groups of games: one 30 s and one 45 s) and one within-subject factor (six measurements of the same individuals over time-repeated measurement). In connection with the assumed aim of our study, the most important is the group*time interaction effect concerning the comparison of changes in the dependent variables (parameters under study) over time in the two groups specified. First, normality of distribution (Shapiro–Wilk test), homogeneity of variance between groups (Levene’s test), and sphericity (Mauchley’s sphericity test, homogeneity of variance of differences between measurements) were checked. The first two conditions were violated in some cases; however, the ANOVA model is quite robust to violations of the normality condition as well as the homogeneity of variance when the sample sizes in groups are equal^32,33^, which is the case in our study. In contrast, the repeated-measure ANOVA model is not robust to violation of the sphericity condition, hence the Greenhouse–Geisser correction for degrees of freedom was applied when this assumption was violated^33^. For significant main effects or interactions, post-hoc comparisons were made using the Bonferroni test. The partial eta-square (η P2) was presented as an effect size measure for main effects and interactions. Analysis was performed in Statistica 13.3.

## Results

Statistical analysis showed no significant differences in the mean values of age (SEP1 = 17.2 ± 0.88 years; SEP2 = 17.1 ± 0.75 years; t = 0.2735, p ≥ 0.05) and training experience (SEP1 = 8.6 ± 3.35 years; SEP2 = 8.7 ± 3.15 years; t = 0.0688, p ≥ 0.05) between the players from both examined groups. Also, in the case of the anthropometric characteristics of the examined athletes, no statistically significant differences were found between the groups (body height: SEP1 = 177.9 ± 5.08 cm; SEP2 = 178.4 ± 4.92 cm; t = 0.2236, p ≥ 0.05; body mass: SEP1 = 70.5 ± 5.55 kg; SEP2 = 71.2 ± 5.31 kg; t = 0.2882, p ≥ 0.05).

Table 2 presents descriptive statistics as well as between group and SSG bout (game number) differences in the values of indicators characterizing the volume of ETL (i.e., TD, PL, and ACC/DEC).

**Table 2:** Descriptive statistics (means and standard deviations) for Player Load, total distance covered, accelerations, and decelerations and a comparison of the levels of these variables according to the game number and the tested group.

Variable	SSG group	Game number					
		1	2	3	4	5	6
PL [a.u.]	SEP1	14.05^1,2,3,4^ (0.96)	13.42^5,6,7,8^ (1.05)	11.97^1,5,9,10,19^ (0.85)	11.27^2,6,11,20^ (1.07)	10.51^3,7,9,21^ (1.27)	9.27^4,8,10,11,22^ (1.19)
	SEP2	17.18^12,13,14^ (2.59)	16.49^15,16^ (2.85)	16.11^17,19^ (2.42)	15.77^12,18,20^ (2.51)	14.90^13,15,21^ (2.01)	13.77^14,16,17,18,22^ (2.28)
	Total	15.62^a,b,c,d^ (2.49)	14.95^e,f,g,h^ (2.62)	14.04^a,e,i,j^ (2.76)	13.52^b,f,k^ (2.98)	12.71^c,g,i,l^ (2.78)	11.52^d,h,j,k,l^ (2.91)
TD [m]	SEP1	79.60 (4.18)	76.81 (4.12)	76.26 (5.65)	69.00 (5.53)	67.08 (5.51)	63.50 (5.06)
	SEP2	103.36 (7.11)	103.28 (6.97)	99.48 (5.78)	97.38 (5.74)	94.07 (7.82)	88.68 (4.96)
	Total	91.48^a,b,c,d^ (13.44)	90.04^e,f,g^ (14.67)	87.87^a,h,i,j^ (13.15)	83.19^b,e,h,k^ (15.56)	80.57^c,f,i,l^ (15.33)	76.09^d,g,j,k,l^ (13.81)
ACC [n]	SEP1	5.00 (0.67)	4.70 (0.48)	4.50 (0.53)	4.10 (0.57)	3.70 (0.48)	3.10 (0.32)
	SEP2	6.90 (1.10)	6.60 (0.97)	6.40 (0.84)	5.90 (0.74)	5.70 (0.67)	5.20 (0.79)
	Total	5.95^a,b,c,d^ (1.32)	5.65^e,f,g^ (1.23)	5.45^a,h,i^ (1.19)	5.00^b,e,j^ (1.12)	4.70^c,f,h,k^ (1.17)	4.15^d,g,i,j,k^ (1.23)
DEC [n]	SEP1	4.60 (0.52)	4.30 (0.48)	4.20 (0.42)	3.80 (0.42)	3.20 (0.42)	2.90 (0.32)
	SEP2	6.50 (1.08)	6.20 (1.13)	6.00 (0.82)	5.80 (1.03)	5.00 (0.94)	4.70 (0.67)
	Total	5.55^a,b,c^ (1.28)	5.25^d,e^ (1.29)	5.10f.^,g^ (1.12)	4.80^a,h,i^ (1.28)	4.10^b,d,f,h^ (1.17)	3.80^c,e,g,i^ (1.06)

For PL: p < 0.05—5, 9, 12, e, p < 0.01—15, 19, 21, p < 0.001—1–4, 6–8, 10, 11, 13, 14, 16–18, 20, 22, a-d, f-l.

For TD: p < 0.05—a, p < 0.001—b-l.

For ACC: p < 0.05—a, k, p < 0.01—e, p < 0.001—b-d, f-j.

For DEC: p < 0.01—h, p < 0.001—a-g, i

^#^The same letter or number for two compared means indicates a statistically significant difference.

For the significant interaction effect group*time, detailed differences are marked using numerical values, while differences for significant main effects of time (“total” row) are marked using letters.

**Figure 2:**
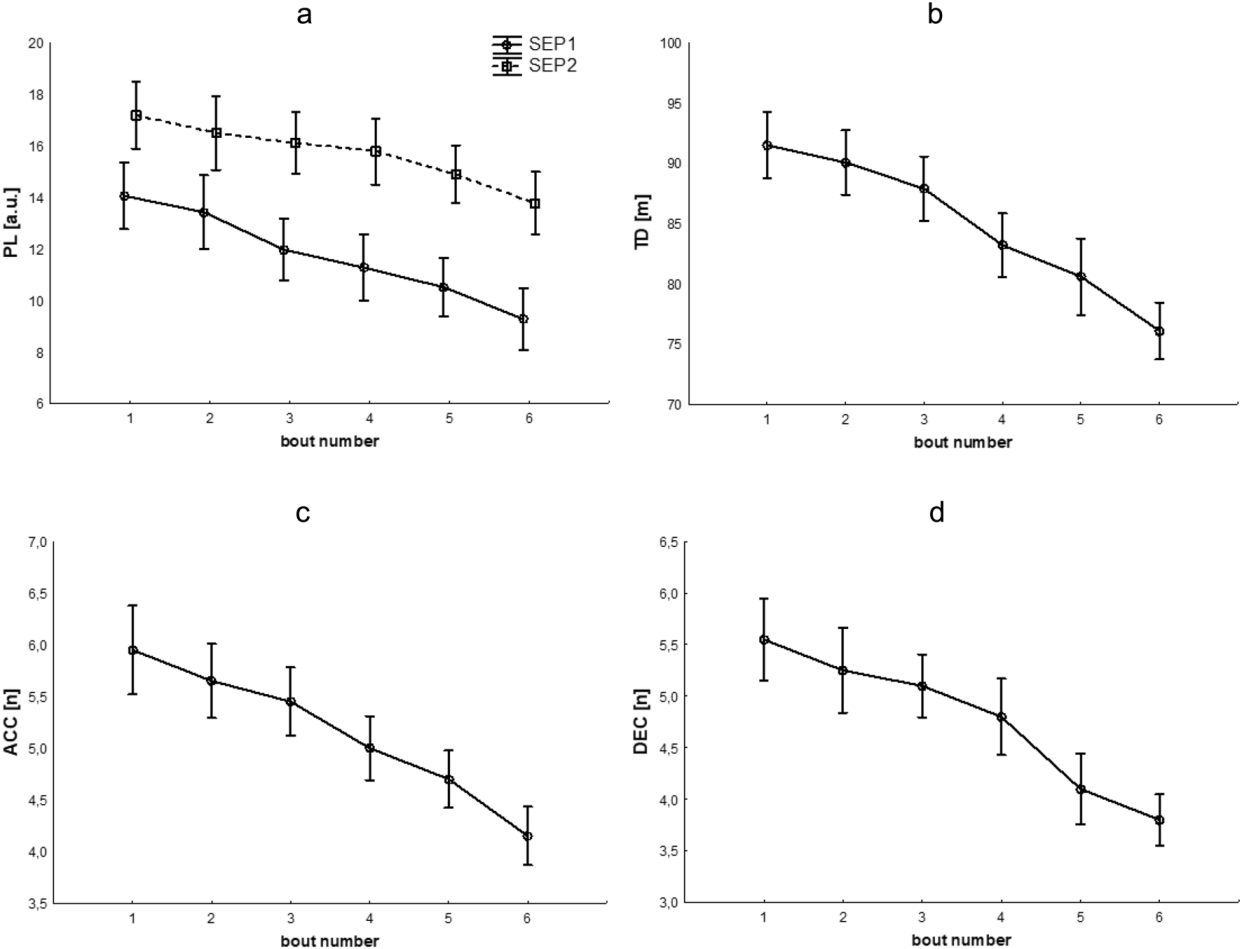
Changes in the level of the tested variables. The graphs show significant changes in Player Load according to the group studied (**a**) and significant overall changes in total distance covered (**b**), number of accelerations (**c**), and decelerations (**d**). PL: player load; TD: total distance covered; ACC: number of accelerations; DEC: number of decelerations.

A significant group*time interaction effect was noted for Player Load (F(5.90) = 2.99, p < 0.05, η P2 = 0.14), indicating a difference in the course of change across groups (Fig. 2a). A greater decrease was found in the PL variable in group SEP1 than in group SEP2. Further, in the SEP1 group, significant decreases in PL values compared to the first measurement were found in the third game. In the SEP2 group, on the other hand, significant decreases in values compared to the first measurement were found beginning in the fourth game. Additionally, from measurements 3 to 6, statistically significantly higher PL values were noted in the SEP2 group compared to SEP1. Statistically significant main effects of group (F(1,18) = 26.40, p < 0.001, η P2 = 0.59) and time (F(5,90) = 58.16, p < 0.001, η P2 = 0.76) were also found. Detailed comparisons of means (post-hoc tests) for the interaction effect and the main effect of time for the variables PL, TD, total ACC, and total DEC can be found in Table 2.

For TD covered, we found no statistically significant group*time interaction effect (F(3.24,58.33) = 1.85, p > 0.05, ε = 0.65). However, there was a significant overall decrease in TD values during games as indicated by a statistically significant main effect of time (F(3.24,58.33) = 67.17, p < 0.001, η P2 = 0.79, ε = 0.65) (Fig. 2b). Significant decreases in values relative to the measurement in the first game begin with the measurement in the third game. A significant group main effect was also noted (F(1,18) = 132.62, p < 0.001, η P2 = 0.88), where the SEP2 group had overall higher TD values than the SEP1 group.

For total ACC, we found no statistically significant group*time interaction effect (F(5,90) = 0.21, p > 0.05). However, a main effect of time was noted (F(5,90) = 34.07, p < 0.001, η P2 = 0.65), indicating a significant overall decrease in the parameter under study (Fig. 2c). Significant decreases in values relative to the measurement in the first game began with the measurement in the third game. Further, a significant group main effect was also noted (F(1,18) = 64.08, p < 0.001, η P2 = 0.78), where the SEP2 group had overall higher total ACC values than the SEP1 group.

For total DEC, we found no significant group*time effect (F(3.28,58.95) = 0.12, p > 0.05, ε = 0.66). In contrast, a main effect of time was noted (F(3.28,58.95) = 33.14, p < 0.001, η P2 = 0.65, ε = 0.66) (Fig. 2d). Significant decreases in values relative to the measurement in the first game begin with the measurement in the fourth game. Further, a significant group main effect was also noted (F(1,18) = 54.38, p < 0.001, η P2 = 0.75), where the SEP2 group had overall higher total DEC values than the SEP1 group.

Figure 2 presents significant interaction effects of group*time (Fig. 2a) or significant main effects of time (Fig. 2b–d) when the interaction effects did not reach statistical significance.

Descriptive statistics and statistical analyses of indicators characterizing external intensity measures (TD/min, PL/min, ACC/min, DEC/min, and MV) are presented in Table 3.

**Table 3:** Descriptive statistics (means and standard deviations) for total distance per minute, Player Load per minute, accelerations per minute, decelerations per minute, maximum velocity, and a comparison of the levels of these variables according to the game number and tested group.

Variable	SSG group	Game number					
		1	2	3	4	5	6
PL/min [a.u.]	SEP1	28.11^1,2,3,4,18^ (1.91)	26.84^5,6,7,8,19^ (2.11)	23.94^1,5,9,10^ (1.70)	22.54^2,6,11^ (2.13)	21.03^3,7,9,12^ (2.55)	18.54^4,8,10,11,12^ (2.39)
	SEP2	22.91^13,14,18^ (3.45)	21.98^15,19^ (3.81)	21.48^16^ (3.23)	21.03^17^ (3.35)	19.86^13^ (2.68)	18.37^14,15,16,17^ (3.04)
	Total	25.51^a,b,c,d^ (3.81)	24.41^e,f,g,h^ (3.89)	22.71^a,e,i,j^ (2.81)	21.78^b,f,k,l^ (2.84)	20.45^c,g,i,k,m^ (2.62)	18.46^d,h,j,l,m^ (2.66)
TD/min [m]	SEP1	159.20^1,2,3,19^ (8.35)	153.63^4,5,6,20^ (8.23)	152.51^7,8,9,21^ (11.30)	138.00^1,4,7,10^ (11.05)	134.16^2,5,8^ (11.01)	127.00^3,6,9,10^ (10.12)
	SEP2	137.82^11,12,13,19^ (9.47)	137.70^14,15,16,20^ (9.29)	132.64^17,21^ (7.70)	129.84^11,14,18^ (7.65)	125.42^12,15^ (10.43)	118.24^13,16,17,18^ (6.61)
	Total	148.51^a,b,c,d^ (14.00)	145.66^e,f,g^ (11.82)	142.57^a,h,i,j^ (13.88)	133.92^b,e,h,k^ (10.16)	129.79^c,f,i,l^ (11.36)	122.62^d,g,j,k,l^ (9.46)
ACC/min [n]	SEP1	10.00^1,2,3^ (1.33)	9.40^4,5^ (0.97)	9.00^6,7^ (1.05)	8.20^1,8^ (1.13)	7.40^2,4,6^ (0.97)	6.20^3,5,7,8^ (0.63)
	SEP2	9.20^9,10,11^ (1.47)	8.80^12^ (1.29)	8.53^13^ (1.12)	7.87^9^ (0.98)	7.60^10^ (0.90)	6.93^11,12,13^ (1.05)
	Total	9.60^a,b,c,d^ (1.42)	9.10^e,f,g^ (1.15)	8.77^a,h,i^ (1.09)	8.03^b,e,j^ (1.05)	7.50^c,f,h,k^ (0.91)	6.57^d,g,i,j,k^ (0.92)
DEC/min [n]	SEP1	9.20 (1.03)	8.60 (0.97)	8.40 (0.84)	7.60 (0.84)	6.40 (0.84)	5.80 (0.63)
	SEP2	8.67 (1.44)	8.27 (1.51)	8.00 (1.09)	7.73 (1.38)	6.67 (1.26)	6.27 (0.90)
	Total	8.93^a,b,c^ (1.25)	8.43^d,e^ (1.25)	8.20f.^,g^ (0.97)	7.67^a,h,i^ (1.11)	6.53^b,d,f,h^ (1.05)	6.03^c,e,g,i^ (0.79)
MV [km∙h^-1^]	SEP1	17.44 (1.94)	17.44 (1.38)	17.30 (1.17)	17.39 (1.46)	17.25 (1.49)	17.34 (1.26)
	SEP2	17.29 (1.61)	17.24 (1.86)	17.47 (2.47)	17.28 (1.50)	17.50 (1.59)	17.36 (1.87)
	Total	17.37 (1.74)	17.34 (1.59)	17.38 (1.88)	17.34 (1.44)	17.38 (1.51)	17.35 (1.56)

For TD/min: p < 0.05—11, 14, 20, p < 0.01—19, 21, a, p < 0.001—1–10, 12, 13, 15–18, b-l.

For PL/min: p < 0.05—18, 19, k, p < 0.01—12, 17, e, p < 0.001—1–11, 13–16, a-d, f-j, l, m

For ACC/min: p < 0.05—9, a, p < 0.01—6, 10, 13, k, p < 0.001—1–5, 7, 8, 11, 12, b-j.

For DEC/min: p < 0.001—a-i,

^#^The same letter or number for two compared means indicates a statistically significant difference.

For the significant interaction effect group*time, detailed differences are marked using numerical values, while differences for significant main effects of time (“total” row) are marked using letters.

**Figure 3:**
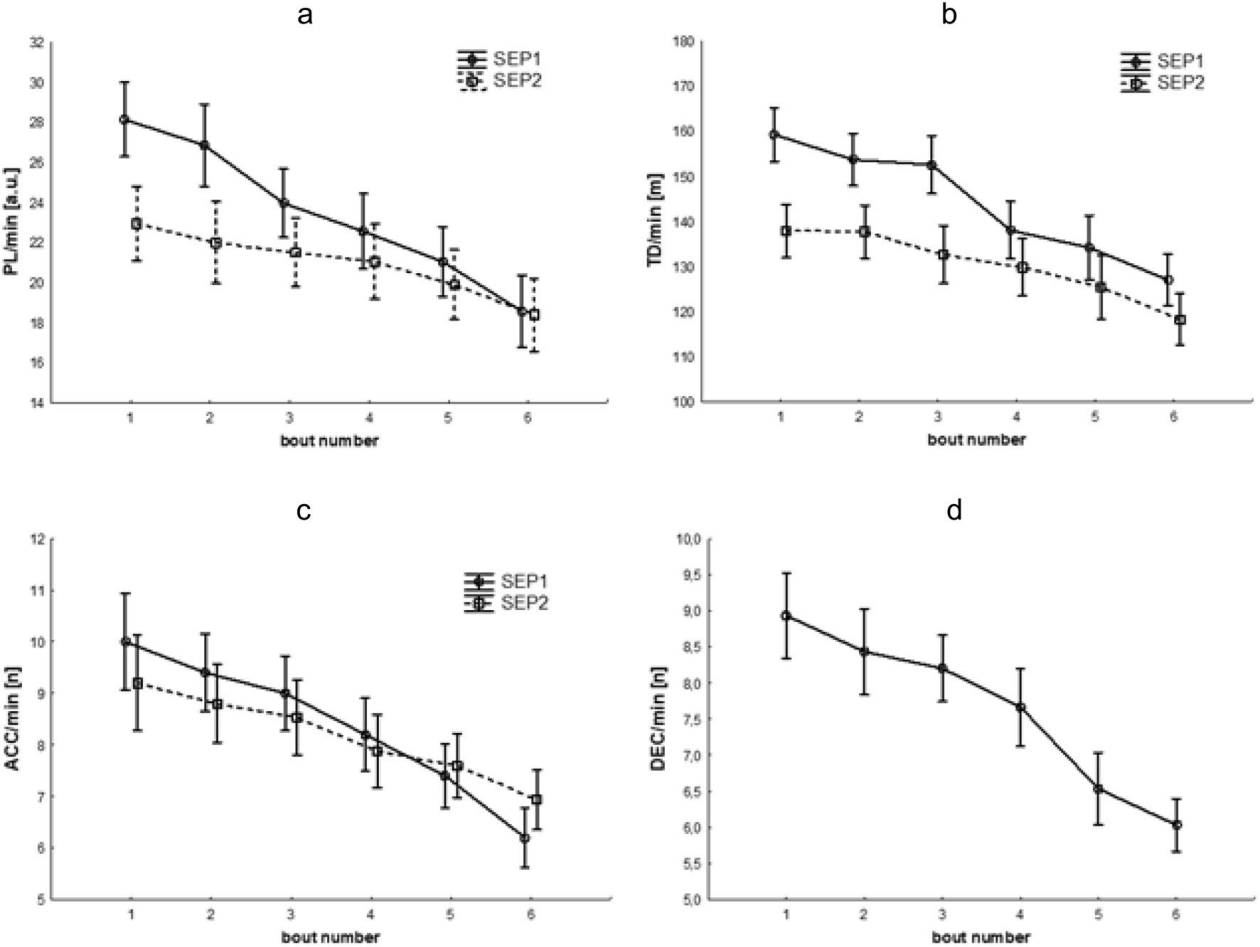
Changes in the level of the tested variables. The graphs show significant changes in Player Load per minute (**a**), total distance per minute (**b**), number of accelerations per minute (**c**) depending on the group studied, and significant overall changes in the number of decelerations per minute (**d**). PL/min: Player Load per minute; TD/min: total distance per minute; ACC/min: number of accelerations per minute; DEC/min: number of decelerations per minute.

A statistically significant group*time interaction effect was noted for PL/min (F(5,90) = 11.32, p < 0.001, η P2 = 0.39), indicating that the study groups differed significantly in changes over time (Fig. 3a). We also found a greater decrease in PL/min in group SEP1 than in group SEP2. In the SEP1 and SEP2 groups, significant decreases in values from the first measurement begin in the third and fifth games, respectively. Statistically significant intergroup differences were also noted in the first two games, where the SEP1 group had higher PL/min levels. We also found statistically significant main effects of group (F(1,18) = 5.34, p < 0.05, η P2 = 0.23) and time (F(5,90) = 72.00, p < 0.001, η P2 = 0.80). Detailed comparisons of means (post-hoc tests) for the interaction effect and the main effect of time for the variables TD/min, PL/min, ACC/min, DEC/min, and MV can be found in Table 3.

A statistically significant group*time interaction effect was noted for TD/min (F(3.43,61.73) = 7.72, p < 0.001, η P2 = 0.30, ε = 0.69), indicating that the study groups differed significantly in changes over time (Fig. 3b). The SEP1 group showed a greater decrease in TD/min than the SEP2 group. In both studied groups, significant decreases in values relative to the first measurement begin in the fourth game. Further, statistically significant intergroup differences in measurements were found during the first three games, where the SEP1 group had higher TD/min levels. We also noted statistically significant main effects of group (F(1,18) = 13.91, p < 0.01, η P2 = 0.44) and time (F(3.43,61.73) = 86.02, p < 0.001, η P2 = 0.83, ε = 0.69).

A statistically significant group*time interaction effect was noted for the number of ACC/min (F(5,90) = 2.59, p < 0.05, η P2 = 0.13), indicating that the study groups differed significantly in changes over time (Fig. 3c). The SEP1 group showed a greater decrease in ACC/min than the SEP2 group. In both groups of players, significant decreases in values compared to the first measurement began in the fourth game. Although no statistically significant main effect of group (F(1,18) = 0.33, p > 0.05) was found, a significant effect of time was obtained (F(5,90) = 39.49, p < 0.001, η P2 = 0.69).

We found no statistically significant interaction effect of group*time for the number of DEC/min (F(5,90) = 1.20, p > 0.05). In contrast, a significant main effect of time was shown (F(5,90) = 36.91, p < 0.001, η P2 = 0.67) (Fig. 3d). Thus, a significant overall decrease in the value of this parameter was found, which, compared to the first measurement, started in the fourth game. However, we found no statistically significant main group effect (F(1,18) = 0.04, p > 0.05).

On the other hand, for MV, we found no significant changes by game type nor significant differences overall (interaction effect of group*time: F(5,90) = 0.18, p > 0.05, effect of time: F(5,90) = 0.01, p > 0.05, group effect: F(1,18) = 0.01,p > 0.05).

Figure 3 presents significant group*time interaction effects (Fig. 3a–c) or significant main effects of time (Fig. 3d) when the interaction effects did not achieve statistical significance.

## Discussion

This was the first study to compare the ETL and its temporal changes during SEP training in the form of 1 × 1 SSGs with different bout durations in elite youth soccer players. Our study revealed a significant overall decrease in the values of measured ETL indicators during SSGs. Furthermore, a greater decrease in the external intensity measures was observed in the 30-s games than in the 45-s games.

Similar to previous studies by other authors^34,35^, we also observed that higher values ​concerning the volume of ETL (TD, PL, total number of ACC and DEC) and lower values of external intensity measures (TD/min, PL/min, ACC/min, and DEC/min) characterize SSG with longer bout duration. However, contrary to our research, Köklü et al.^34^ and Alcântara et al.^35^ did not consider ETL in the context of SEP training. As shown by Ade et al.^19^, ETL in SEP training is affected by the form of the training. This author measured higher TD, very-high-speed running distance, and sprint distance in SEP running drills compared to SEP 1 × 1 SSG training. On the other hand, SEP 1 × 1 SSG training was characterized by higher values of high ACC and DEC distance at 2–3 m·s^−2^ and maximum DEC distance at < –3 m·s^−2^ compared to SEP generic running training. A larger acute effect of running drills on players’ ETL variables, excluding distance performed at high DEC compared to SSG was also shown by Castagna et al.^36^. In his later research, comparing position-specific speed endurance soccer drills in production vs maintenance training, Ade et al.^37^ showed that ETL is also affected by different recovery periods between bouts (60 s vs 150 s in SEM and SEP training, respectively). These authors observed significantly higher values of TD (5%) and distance covered in high-speed running (12%), very-high-speed running (49%), and sprinting (218%) in the SEP protocol compared to the SEM protocol. In this comparison, no differences were noted for acceleration and deceleration demands. Similar results were also obtained in a study comparing acute responses after SEP and SEM training carried out in the form of SSG and running drills^36^. Overall, a longer recovery time in SEP training (150 s vs 60 s in SEM protocols) led to more pronounced responses in players’ ETL.

Speed endurance training (SET) should provide a physical and physiological overload stimulus that will increase anaerobic capacity and resistance to fatigue related to anaerobic soccer match demands^14,15,17^. An athlete’s physical stimulation during training is measured by various ETL metrics. Maintaining the values of these indicators at a relatively constant level during subsequent SSGs or intermittent running repetitions guarantees an appropriate physical and physiological response to the applied training stimulus. Conducting SET in the form of interval runs or specific soccer running drills does not encounter this problem because it is assumed that each competitor must maintain a specific work output in a specific time and form. However, primarily intermittent running does not improve specific technical and tactical abilities as well as specific motor skills related to the large number of accelerations, decelerations, and changes of direction found in SSGs^19^. Using SSG as a soccer-specific form of SET, however, we must bear in mind the possibility of a decrease in the work performed by the player due to less restrictive possibilities of its control.

Despite the mentioned benefits of using SSGs in SET, no research on temporary changes in work performed across repetitions during short-bout SSGs was found in the available literature. Previous studies directly or indirectly addressed the problem of temporary changes in the efficiency of work performed in SSGs with longer than 3-min bout duration and with the use of a larger number of players (3 × 3, 4 × 4, and 5 × 5)^35,38–44^. The studies mentioned above showed changes in indicators such as PL, TD, ACC, or DEC in SSG training but in a different time frame and format than the one used in the SEP method.

An analysis of time changes in the effectiveness of work performed in the SEP training was carried out by Ade et al.^37^. However, the researchers did not use the SSG protocol; instead, they studied eight 30-s bouts of position-specific speed endurance soccer drills. When they compared their results to the measurements from the first repetition, lower TD values were observed in repetition 7, very-high-speed running distance in repetitions 6, 7, and 8, and peak speed in repetitions 7 and 8. Interestingly, this study showed that the use of the same load protocol but with a shortened recovery period (from 150 s in SEP to 60 s in SEM training) resulted in a highly significant increase in the coefficient of variation (CV) across all speeds, with the lowest CVs evident for TD and the highest CVs for sprinting. These findings, in turn, show lower variations of ETL measures across repetitions in SEP protocols.

In our research, as in Ade et al.^37^, we observed overall decreases in the values ​​of the studied ETL parameters, except for MV, measured across six repetitions in both SSG groups. Compared to Ade et al.^37^, in our study, significant declines in ETL values relative to the measurement in the first set were found beginning in the third (PL and PL/min in SEP1 group, TD, ACC in both groups) or fourth (PL in SEP1 group, DEC, TD/min, ACC/min, and DEC/min in both groups) repetition. These differences were probably due to the unstructured nature of SSGs involving other players, which was used in our study, and the lower control of exercise intensity compared to exercises performed in isolation by a single player (i.e., Ade et al.^37^).

The differences between the studies may also have resulted from a different work-to-rest ratio (1:4 in our research and 1:5 in Ade et al.’s^37^ work). Moreover, in our study, ETL indicators were divided into external volume and intensity measures. In the case of external volume measures in group SEP1, a greater variation across repetitions than in group SEP2 was found only in the PL values. In the case of external intensity measures, intergroup differences during changes across repetitions concerned the values ​​of TD/min, PL/min, and ACC/min. Also, in this case, a greater decrease was noted in the group of 30-s bout durations in comparison with the group of 45-s repetitions.

Interestingly, Ade et al.^37^ noted greater decreases in ETL occurred in the SEM group, which was characterized by a greater cardiovascular load (greater mean %HRmax and peak %HRmax) but a lower stimulation of anaerobic metabolism than in the SEP group (lower blood lactate post drill). Further, the SEP protocol, which was characterized by a higher ETL and a smaller decrease in ETL across repetitions compared to the SEM protocol, caused greater acute fatigue immediately after training. Concerning different bout duration SSG protocols in SEP training, in our previous work comparing the physiological response to this type of training^26^, we showed that both of these protocols elicit a similar cardiovascular and metabolic response measured by mean %HRmax and blood lactate. However, in the 30-s protocol, compared to the 45-s games, we showed deeper acid–base imbalances (greater decrease in blood HCO_3_^-^ and base excess level during SSG repetitions, and no return of pH values after 30 min of rest to baseline values in the SEP1 group).

In summary, even though the mechanisms of drops in intensity and volume of external training loads are not fully explained, the use of SSGs in SEP training is associated with a temporary reduction in the effectiveness of work performed across repetitions. The form of training, the duration of repetitions and rest breaks as well as the related physiological demands of the training used may all cause various changes in work efficiency measures. Understanding these impacts undoubtedly requires further research.

Despite several important findings, the reader should be aware of the limitations of the present study. First, the research was carried out on small groups of soccer players. In the future it would have been more appropriate to use a crossover design where the two studied groups would have performed all six successive SSGs with different bout duration. Second, baseline fitness levels were not established before beginning the training protocol. In future research, players could complete exercise tests to determine whether their current fitness levels affect ETL across SSG repetitions. Finally, we did not analyze if ETL changes were influenced by the verbal encouragement given by the coaches conducting the training. Further research concerning the use or lack of external motivation of players during training is warranted.

## Conclusions

The use of SSGs in SEP training is associated with a decrease in the effectiveness of work performed across repetitions. Despite maintaining the same work-to-rest ratio (1:4), a greater decrease in ETL is observed in protocols characterized by shorter bout durations and rest breaks. Greater changes were observed in the ETL indicators characterizing external intensity measures than external volume measures; therefore, the more sensitive ETL indicators are those reported in relative terms (per minute).

In practice, it seems that short-bout SSGs used in SEP training, due to their unstructured nature and involving other players, are a training form in which control of exercise intensity may be difficult. For this reason, online ETL control during the short-bout SSG training should be introduced, as data analysis after the end of training may be too late. Finally, due to the decrease in the measured ETL indicators already in the 3rd or 4th repetition, it seems reasonable to increase the work-to-rest ratio from the applied 1:4 to even 1:6. Further, with more than four repetitions in the training, it seems reasonable to use two series of repetitions with a longer rest break between the two sets.

## Data Availability

The datasets generated during and/or analyzed during the current study are available from the corresponding author on reasonable request.
